# Prolonged anesthesia duration is associated with postoperative thyroid hormone reduction in pediatric surgical patients: a retrospective cohort study

**DOI:** 10.3389/fped.2026.1774627

**Published:** 2026-04-15

**Authors:** Mengqin Shan, Chaoyang Tong, Xin Fu, Yuxin Zhang, Liping Sun, Kan Zhang, Jijian Zheng

**Affiliations:** 1 Department of Anesthesiology, Shanghai Children's Medical Center, Shanghai Jiao Tong University School of Medicine, China; 2 Department of Anesthesiology, Zhongshan Hospital, Fudan University, Shanghai, China; 3 Shanghai Jiao Tong University School of Medicine, Shanghai, China

**Keywords:** anesthesia, cardiac surgery, low T3 syndrome, pediatric surgery, retrospective study, thyroid hormone

## Abstract

**Background:**

Postoperative reduction of thyroid hormones (TH), particularly triiodothyronine (T3), is frequently observed but poorly characterized in pediatric surgical patients. This study aimed to identify risk factors for postoperative TH reduction in pediatric patients, analyze the association between anesthetic factors and perioperative TH changes, and explore the relationship between postoperative TH reduction and clinical outcomes in pediatric cardiac surgery patients.

**Methods:**

We conducted a retrospective analysis of 233 pediatric patients (ages 0–18) undergoing surgery and anesthesia at Shanghai Children's Medical Center between 2014 and 2023. Patients were categorized into low thyroid hormone (LTH) and non-LTH groups based on postoperative free T3 (FT3) and/or free thyroxine (FT4) levels below the lower reference limits. Multivariable logistic and ridge regression were used to identify independent predictors. Clinical outcomes were analyzed in a sub-cohort of cardiac surgery patients.

**Results:**

Postoperative LTH occurred in 78 patients (33.5%). Longer anesthesia duration, surgical duration, higher ASAphysical status (III-V), cardiac surgery, and cardiopulmonary bypass (CPB) use were associated with LTH in univariate analysis (*P* < 0.05). Multivariable regression identified anesthesia duration as a significant independent risk factor (OR = 1.335, 95% CI: 1.144–1.557, *P* < 0.001), a finding confirmed by ridge regression. This risk remained consistent regardless of surgery type (*P* for interaction = 0.747) or CPB use (*P* for interaction = 0.826). In an exploratory analysis of cardiac surgery patient, severe TH reduction (low both FT3 and FT4) was significantly associated with longer postoperative hospital stays (*P* = 0.035).

**Conclusion:**

Longer anesthesia duration is independently associated with postoperative TH reduction in children. In cardiac surgery, the degree of this reduction correlates with prolonged hospitalization. These findings highlight anesthesia duration as a potentially risk factor for postoperative thyroid hormone reduction, which may inform perioperative management and warrant further investigation into underlying mechanisms.

## 1 Introduction

Perioperative low triiodothyronine (T3) syndrome, characterized by reduced serum free T3 (FT3) and total T3 levels, with normal or low concentrations of thyroxine (T4) and thyroid-stimulating hormone (TSH), represents a prevalent endocrine dysregulation in the surgical population (1, 2). This condition is particularly frequent in pediatric patients undergoing cardiac surgery with cardiopulmonary bypass (CPB) (3, 4). The pediatric population is uniquely vulnerable due to an immature hypothalamic-pituitary-thyroid (HPT) axis and the essential role thyroid hormones play in neurocognitive development (5, 6). Despite this physiological distinctiveness, the underlying determinants and clinical consequences of perioperative T3 reduction in children remain under-researched.

The mechanisms driving postoperative thyroid hormone reduction are not yet fully understood. Current hypotheses include surgical and inflammatory stress-induced suppression of the HPT axis (7), as well as altered peripheral metabolism mediated by suppressed 5'-deiodinases (D1 and D2) and increased type 3 deiodinase (D3) activity, which promotes the generation of inactive reverse T3 (rT3) (8). Whether anesthesia serves as an independent contributor to this hormonal disturbance remains a critical unresolved question. While animal studies suggest that inhalational anesthetics can significantly lower T3 and T4 levels (9), clinical studies are confounded by the inevitable effects of surgery itself. clinical evidence remains confounded by the concomitant effects of surgical stress.

Clinically, postoperative thyroid hormone reduction is associated with adverse outcomes. In pediatric cardiac surgery, decreased thyroid hormone levels correlate with increased cardiopulmonary complications and delayed recovery, serving as a potential predictive marker for prognosis (10). Evidence from some studies suggests that T3 supplementation may confer benefits on myocardial function and recovery (11–13). However, the absence of large-scale clinical trials assessing the safety and efficacy of such interventions has resulted in a significant gap in evidence-based management.

To address these limitations, we conducted a retrospective study of pediatric surgical patients. The objectives were to: (1) identify risk factors for postoperative thyroid hormone reduction in children; (2) analyze the relationship between anesthetic factors and perioperative thyroid hormone changes; and (3) explore the association between postoperative thyroid hormone reduction and clinical outcomes specifically in pediatric cardiac surgery patients. This study aims to clarify the role of anesthesia in pediatric postoperative thyroid hormone reduction and provide insights into its clinical impact, thereby informing optimized perioperative care for children.

## 2 Methods

### 2.1 Study design and patients

Following the approval of the Institutional Review Board of Shanghai Children's Medical Center (Permission NO: SCMCIRB-K2025115-1). This retrospective study analyzed data from surgical cases performed between 2014 and 2023 at Shanghai Children's Medical Center. Patient inclusion criteria were children (age 0-18 years) hospitalized and undergoing surgery and anesthesia who underwent thyroid function testing within 7 days of surgery. Preoperative presence of abnormal thyroid function, thyroid surgery or neurosurgery, and preterm births were excluded. Eventually 233 children were included in the analysis. Considering the retrospective nature of the study and the anonymity of the data, a formal request has been made for a waiver of informed consent. This article adheres to the Strengthening the Reporting of Observational Studies in Epidemiology (STROBE) guidelines.

### 2.2 Data collection and study outcomes

Data were retrospectively collected from the hospital electronic medical record system. The extracted information included patient demographics (age, sex, height, and weight), American Society of Anesthesiologists (ASA) physical status classification, and pertinent surgical details such as procedure type, anesthetic technique (total intravenous anesthesia, inhalational anesthesia, or combined intravenous-inhalational anesthesia), duration of anesthesia and surgery, specific anesthetic agents used, and whether cardiopulmonary bypass was employed. Laboratory parameters included preoperative and postoperative thyroid function tests (TSH, FT3, and free thyroxine [FT4]) as well as other relevant laboratory investigations.

The primary outcome of this study was postoperative thyroid hormone (TH) levels. Within the defined 7-day postoperative observation window, none of the patients in the study cohort received exogenous thyroxine replacement therapy. Based on postoperative TH measurements obtained within 7 days after surgery, patients were categorized into two groups: the low TH group (LTH) and the non-low TH group (Non-LTH). LTH was defined as postoperative FT3 and/or FT4 levels below the lower limit of the age-specific pediatric reference intervals established by the institutional laboratory. The applied reference intervals for FT3 were as follows: 2.2–7.4 pmol/L (<1 month of age); 3.8–10.0 pmol/L (1 month to <1 year); 3.8–9.8 pmol/L (1 to <14 years); and 3.8–8.0 pmol/L (14 to 18 years). The reference interval for FT4 was 9.10–19.5 pmol/L. These intervals were derived from the assay system routinely used in our institution and may vary across laboratories due to differences in analytical methods and reagents. Among cardiac surgery patients, the LTH group was further subdivided into a Low T3 (LT3) group (isolated low FT3) and a Low T3-Low T4 (LT3-LT4) group (both FT3 and FT4 below the lower reference limits).

The secondary outcome was a composite of adverse clinical outcomes, collected exclusively in the cardiac surgery patient cohort. This composite outcome included: postoperative hospital stay, prolonged ICU stay, delayed extubation, cardiovascular complications, and pulmonary complications. For this analysis, “prolonged ICU stay” was defined as an ICU length of stay exceeding 3 days. “Delayed extubation” was defined as failure to extubate within 24 h post-surgery. “Cardiovascular complications” encompassed arrhythmias, low cardiac output syndrome, reactive pulmonary hypertension, deep venous thrombosis, and the requirement for cardiopulmonary resuscitation. “Pulmonary complications” were defined as the presence of one or more of the following during the hospitalization: pneumonia, hypoxemia, pneumothorax, pleural effusion, or reintubation.

### 2.3 Statistical analysis

There was no statistical power analysis conducted before the study, with the objective of including as many eligible patients as possible. Categorical variables were presented as frequency (percentage). Based on the distribution characteristics, either the chi-square test or Fisher's exact test was employed for comparison, with the latter applied when expected cell counts were <5. For continuous variables, the Shapiro–Wilk test was first performed to assess normality. Normally distributed variables were expressed as mean ± SD (x¯ ± s), while non-normally distributed variables were reported as median with interquartile range [M (P25, P75)]. Between-group comparisons for continuous variables were conducted using the Mann–Whitney U test (two groups) or Kruskal–Wallis H test (multiple groups).

Following the screening of statistically significant variables (*P* < 0.05), a backward stepwise multivariable logistic regression model was built to identify independent risk factors for low postoperative thyroid hormone levels. To evaluate the impact of multicollinearity, diagnostic measures were first applied, including the variance inflation factor (VIF >5) and inter-variable correlation coefficients (Pearson/Spearman |r| > 0.7). Regularized regression (Ridge regression) was used to assess the influence of multicollinearity on the odds ratios (ORs). Finally, subgroup analysis was conducted according to surgery type. All conventional statistical analyses were performed using SPSS 26.0, while Ridge regression was implemented in R 4.4.2 (glmnet package). The significance threshold was uniformly set at *α* = 0.05 (two-tailed test).

## 3 Results

**Figure 1:**
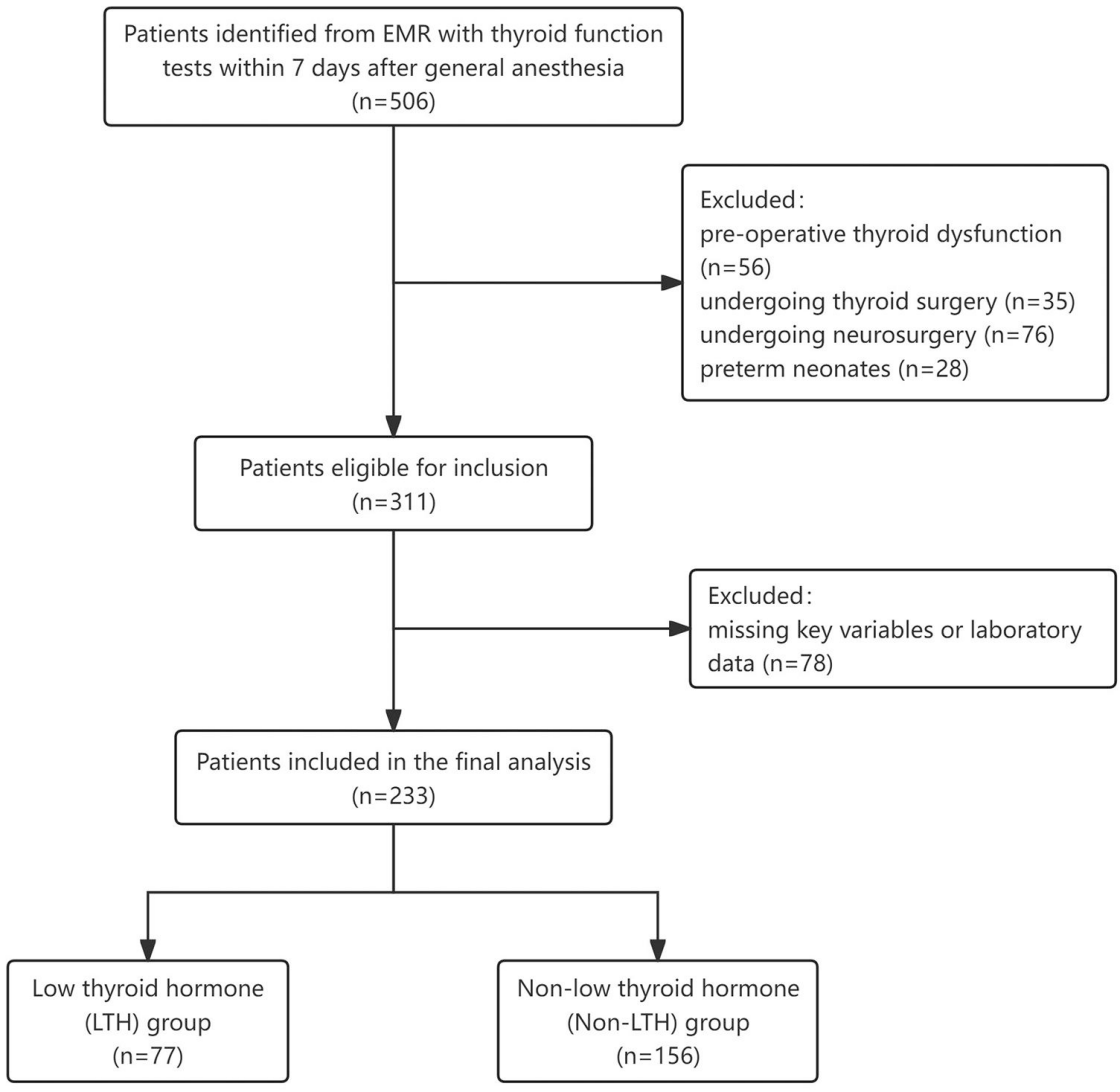
Flow chart of patient enrolment.

A total of 233 patients were included in this study, and the patient enrollment and grouping are detailed in Figure 1. Based on postoperative thyroid hormone levels, the patients were divided into two groups: 78 patients were categorized into the decreased thyroid hormone (LTH) group, while the remaining 156 patients comprised the non-decreased thyroid hormone (Non-LTH) group.

### 3.1 Patient characteristics and univariate analysis

The baseline characteristics of the two groups are summarized in Table 1. There was no significant difference in the median age between the LTH and Non-LTH groups [1.41 years [IQR, 0.33–8.00] vs. 1.62 years [IQR, 0.34–7.03], *P* = 0.779]. Similarly, no significant intergroup differences were observed in terms of sex distribution (55.8% vs. 62.2% male, *P* = 0.353), weight (median 10.0 kg vs. 11.0 kg, *P* = 0.972), or primary anesthetic technique (75.3% vs. 64.7% Intravenous-Inhalation Combined Anesthesia, *P* = 0.257). However, significant differences were identified in key perioperative parameters. The LTH group demonstrated significantly longer anesthesia duration (median 3.16 h vs. 1.83 h, *P* < 0.001) and surgical duration (median 2.25 h vs. 1.42 h, *P* < 0.001). Furthermore, the LTH group had a significantly higher proportion of patients undergoing cardiac surgery (46.8% vs. 28.2%, *P* = 0.005), and a higher rate of cardiopulmonary bypass (CPB) utilization (41.0% vs. 23.1%, *P* = 0.008). The proportion of patients with an ASA physical status classification ≥ III was also higher in the LTH group (91.0% vs. 78.8%, *P* = 0.022).

**Table 1:** Baseline and intraoperative characteristics among groups.

Variable	Non-LTH (*n* = 156)	LTH (*n* = 77)	*p*-value
Age, years	1.62 (0.34, 7.03)	1.41 (0.33, 8.00)	0.779
Sex, *n* (%)			
Male	97 (62.2%)	43 (55.8%)	0.353
Female	59 (37.8%)	34 (44.2%)	
Height, cm	81 (60, 120)	78 (57, 125)	0.883
Weight, kg	11.0 (5.7, 21.4)	10.0 (5.1, 24.0)	0.972
ASA, *n* (%)			0.022*
Ⅰ-Ⅱ	33 (21.2%)	7 (9.0%)	
Ⅲ-Ⅳ	123 (78.8%)	70 (91.0%)	
Type of Surgery, *n* (%)			0.005*
Cardiac surgery	44 (28.2%)	36 (46.8%)	
Non-cardiac surgery	112 (71.8%)	41 (53.2%)	
CPB			0.006*
Yes	36 (23.1%)	31 (40.3%)	
No	112 (71.8%)	46 (59.7%)	
Anesthetic Technique			0.257
TIVA	22 (14.1%)	7 (9.1%)	
Inhalational anesthesia	33 (21.2%)	12 (15.6%)	
Intravenous-Inhalation Combined Anesthesia	101 (64.7%)	58 (75.3%)	
Anesthesia duration, h	1.42 (0.67, 2.47)	2.25 (1.25, 3.46)	<0.001*
Surgical Duration, h	1.83 (0.92, 3.15)	3.16 (2.00, 4.50)	<0.001*

Categorical variables are presented as number (percentage); continuous variables are presented as median (interquartile range).

* Indicates a statistically significant difference (*P* < 0.05). TH: Thyroid Hormone; ASA: American Society of Anesthesiologists; CPB: Cardiopulmonary bypass; TIVA: Total Intravenous Anesthesia.

### 3.2 Multivariable regression analysis

Given the strong association between type of surgery and cardiopulmonary bypass (CPB) (Phi coefficient = 0.859, *P* < 0.001), CPB was retained in the model to avoid redundancy. Multicollinearity was assessed using variance inflation factors (VIF). Most variables showed acceptable values (<5), although anesthesia duration (VIF = 5.560) and surgical duration (VIF = 4.896) suggested potential collinearity, primarily driven by their strong correlation. Both variables were retained due to their clinical relevance, and ridge regression was applied to ensure model robustness. In the multivariable logistic regression analysis, longer anesthesia duration was identified as an independent factor associated with postoperative thyroid hormone reduction [odds ratio [OR] = 1.335, 95% confidence interval [CI]: 1.144–1.557, *P* < 0.001] (Tables 2). This association remained consistent in ridge regression (OR = 1.11, 95% CI: 1.04–1.20), confirming the robustness of the findings.

**Table 2:** Multivariate analysis of predictors for postoperative hypothyroidism.

Variable	OR (95% CI)	*p*-value
ASA (Ⅲ-Ⅳ)	0.557 (0.232–1.437)	0.237
CPB	0.810 (0.403–1.629)	0.555
Surgical Duration	0.956 (0.675–1.353)	0.799
Anesthesia duration	1.335 (1.144–1.557)	<0.001*

ASA, American Society of Anesthesiologists; CPB, Cardiopulmonary bypass.

* Indicates a statistically significant difference (*P* < 0.05).

### 3.3 Subgroup analyses

**Figure 2:**
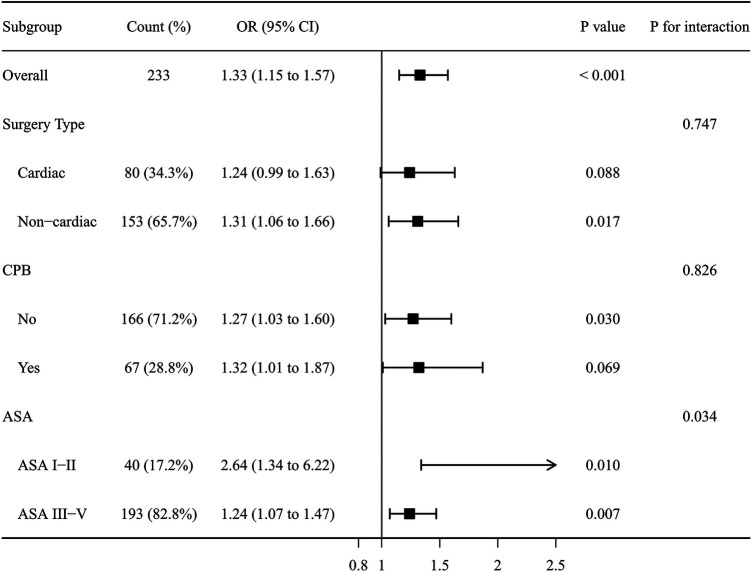
Subgroup analysis of the association between anesthesia duration and postoperative low thyroid hormone levels. ASA: American Society of Anesthesiologists; CPB: Cardiopulmonary bypass.

To evaluate the robustness of the association between anesthesia duration and postoperative low thyroid hormone (LTH) levels, stratified subgroup analyses with interaction tests were performed (Figure 2). Crucially, interaction tests indicated no significant heterogeneity regarding surgery type (*P* for interaction = 0.747) or the application of cardiopulmonary bypass (CPB) (*P* for interaction = 0.826). Although the association reached statistical significance in the non-cardiac subgroup (OR = 1.31, 95% CI: 1.06–1.66, *P* = 0.017) but not in the cardiac subgroup (OR = 1.24, 95% CI: 0.99–1.63, *P* = 0.088), the direction and magnitude of the estimated risk were highly consistent across these distinct surgical cohorts. Furthermore, prolonged anesthesia duration remained a significant risk factor regardless of baseline physical status. A statistical interaction was observed for ASA classification (*P* for interaction = 0.034), with the association remaining significant in both the ASA I-II (OR = 2.64, 95% CI: 1.34–6.22, *P* = 0.010) and ASA III-IV strata (OR = 1.24, 95% CI: 1.07–1.47, *P* = 0.007).

### 3.4 Association between thyroid hormone alteration and postoperative clinical outcomes

To explore the potential association between postoperative thyroid hormone reduction and clinical outcomes, we analyzed a subgroup of patients undergoing cardiac surgery. The final cohort included 64 patients after excluding those with missing outcome data. Baseline characteristics were comparable between the LTH (*n* = 27) and Non-LTH (*n* = 37) groups (Supplementary Table S1).

No statistically significant differences were observed between the two groups in delayed extubation or cardiovascular complications (Table 3). Although patients in the LTH group tended to have longer postoperative hospital stay, prolonged mechanical ventilation, extended ICU stay, and a higher incidence of pulmonary complications, these differences did not reach statistical significance.

**Table 3:** Postoperative outcome indicators of patients undergoing cardiac surgery.

Variable	Non-LTH (*n* = 37)	LTH (*n* = 27)	*p*-value
Postoperative hospital stay, days	11 (8–18)	15 (10–21)	0.064
ICU length of stay, hours	68 (39–185)	115 (66–257)	0.136
Mechanical ventilation, hours	24 (6–80)	42 (22–97)	0.159
Prolonged ICU stay	18 (48.6%)	19 (70.4%)	0.082
Delayed extubation	18 (48.6%)	16 (59.3%)	0.401
Cardiovascular complications	12 (32.4%)	9 (31.0%)	0.940
Pulmonary complications	4 (10.8%)	7 (25.9%)	0.113

In an exploratory analysis, patients with reduced thyroid hormone levels were further stratified into low T3 (LT3) and combined low T3-low T4 (LT3-LT4) groups. A difference in postoperative hospital stay was observed among the three groups (*P* = 0.035) (Table 4). However, given the limited sample size and exploratory nature of this analysis, these findings should be interpreted with caution. A similar increasing trend in pulmonary complications was noted with greater severity of thyroid hormone reduction, although this did not reach statistical significance.

**Table 4:** Postoperative outcome indicators of patients undergoing cardiac surgery.

Variable	Non-LTH (*n* = 37)	LT3 (*n* = 23)	LT3-LT4 (*n* = 4)	*p*-value
Postoperative hospital stay, days	11 (8–18)	14 (10–19)	22 (19–37)	0.035*
ICU length of stay, hours	68 (39–185)	112 (66–205)	200 (68–446)	0.296
Mechanical ventilation, hours	24 (6–80)	40 (21–93)	70 (27–144)	0.252
Prolonged ICU stay	18 (48.6%)	16 (69.6%)	3 (75.0%)	0.216
Delayed extubation	18 (48.6%)	13 (56.5%)	3 (75.0%)	0.556
Cardiovascular complications	12 (32.4%)	8 (34.8%)	1 (25.0%)	0.926
Pulmonary complications	4 (10.8%)	5 (21.7%)	2 (50.0%)	0.110

* Indicates a statistically significant difference (*P* < 0.05).

## 4 Discussion

Our findings indicate that longer anesthesia duration, longer surgical duration, a higher ASA (III-V), and CPB are potential risk factors for postoperative thyroid hormone reduction. Crucially, multivariable analysis confirmed that anesthesia duration was independently associated with this condition. Furthermore, the degree of thyroid hormone reduction was correlated with a longer postoperative hospital stay.

Existing evidence suggests that the duration of CPB is correlated with the severity of postoperative thyroid hormone alterations, characterized by decreased FT3 and FT4 and elevated rT3 levels. Potential mechanisms include hemodilution, hypothermia, and ischemia-reperfusion injury during CPB, which may collectively contribute to the development of low T3 syndrome. However, Velissaris et al. reported no significant difference in FT3 changes between patients undergoing off-pump cardiac surgery and those with CPB, suggesting that CPB may not be the sole trigger for euthyroid sick syndrome (ESS) in cardiac surgical patients (14, 15). In our study, a significant association between longer anesthesia duration and reduced thyroid hormone levels was observed even in the non-cardiac surgery subgroup, implying that anesthetic exposure may disrupt thyroid homeostasis through mechanisms independent of CPB.

A key consideration in this study is the collinearity between anesthesia duration and surgical duration, as prolonged procedures inherently involve both extended anesthesia exposure and greater surgical stress. To mitigate concerns regarding this interdependence, we conducted robustness checks using ridge regression and performed subgroup analyses. Both approaches consistently indicated that anesthesia duration retained an independent association with postoperative thyroid hormone reduction. These findings enhance the robustness of our primary results and support the notion of an independent effect of anesthesia duration, distinct from that of surgical duration.

To explore the potential association between postoperative TH reduction and clinical outcomes, we focused on a sub-cohort of cardiac surgery patients to minimize heterogeneity in surgical type. Our analysis revealed that the degree of postoperative TH reduction was associated with prolonged hospital stay; however, no statistically significant differences were observed in ICU length of stay or cardiovascular complications between the LTH and Non-LTH groups. These findings differ from those reported by Plumpton et al., who identified postoperative low T3 as a significant predictor of delayed recovery, low cardiac output, and mortality in surgical patients (16, 17). This discrepancy may partly reflect the relatively small sample size in our cardiac surgery subgroup, which limited the statistical power to detect significant associations. Importantly, given the exploratory nature of this analysis and the limited sample size, these findings should be interpreted with caution. Further studies with larger and more homogeneous cohorts are warranted to better clarify the potential prognostic role of postoperative TH reduction.

The duration of postoperative thyroid hormone reduction is likely influenced by factors such as surgical type and anesthesia duration. In children undergoing corrective surgery for congenital heart disease (CHD), thyroid hormone levels decline markedly within the first 24 h, reach a nadir at 24–48 h, and gradually recover thereafter, although remaining below preoperative levels for several days. While most children return to their preoperative levels by postoperative day 7, those with complex CHD tend to experience more profound and prolonged suppression of thyroid function (3, 4, 18). In the present study, the retrospective design limited the completeness of thyroid function data. Although patients with known thyroid dysfunction were excluded, baseline measurements were unavailable in some cases. In addition, postoperative assessment was based on a single measurement within 7 days after surgery, which precluded evaluation of dynamic changes and may have failed to capture the absolute nadir in all patients. This limitation may have led to an underestimation of the true extent of thyroid hormone suppression.

This study suggests an association between longer anesthesia duration and postoperative thyroid hormone reduction; however, several limitations should be acknowledged. First, the retrospective cohort design inherently limits the control for confounding factors. Potential selection and information biases, along with unmeasured residual confounders not captured in our data, may have influenced the accuracy of the effect estimates and preclude a comprehensive assessment of the relationship between anesthesia and thyroid hormone levels. Second, the relatively small sample size may affect the robustness and statistical power of our findings. Furthermore, as a single-center observational study, the case mix of surgical types and choices of anesthetic protocols were influenced by local practice guidelines, which may limit the generalizability of our findings to other populations and clinical settings. Future research should involve larger, prospective, multi-center studies designed to better control for confounding variables to fully elucidate the impact of anesthesia on thyroid function. Through multidisciplinary collaboration and further investigation, these efforts hold the potential to inform new strategies for enhancing anesthetic safety and improving patient outcomes

## 5 Conclusion

Anesthesia duration is an independent risk factor for postoperative thyroid hormone reduction in pediatric patients under general anesthesia. In cardiac surgery, severe reduction correlates with prolonged hospitalization. Recognizing this association may refine perioperative risk assessment and guide individualized management strategies.

## Data Availability

The raw data supporting the conclusions of this article will be made available by the authors, without undue reservation.
