# Supplementary material for: Prolonged anesthesia duration is associated with postoperative thyroid hormone reduction in pediatric surgical patients: a retrospective cohort study
Source: Front Pediatr. 2026 Apr 15;14:1774627. doi: 10.3389/fped.2026.1774627 (PMC13125116; doi:10.3389/fped.2026.1774627)
Supplement: Supplementary file 1 [file Table1.docx]

| Variable | Non-LTH  (n=37) | LTH  (n=27) | p-value |
| --- | --- | --- | --- |
| Sex, n (%) |  |  | 0.814 |
| Male | 23 (59.3%) | 16 (59.3%) |  |
| Female | 14 (40.7%) | 11 (40.7%) |  |
| Age, years | 0.68 (0.18，1.70) | 0.34 (0.26，2.21) | 0.708 |
| Hight, cm | 68 (56，80) | 58 (55，80) | 0.337 |
| Weight, kg | 7.25 (4.50，9.68) | 6.20 (4.20，11.20) | 0.908 |
| ASA, n (%) |  |  | / |
| Ⅰ-Ⅱ | 0 (00.0%) | 0 (00.0%) |  |
| Ⅲ-Ⅴ | 37 (100%) | 27 (100.0%) |  |
| Complex cardiac surgery, n (%) |  |  | 0.905 |
| Yes | 17 (45.9%) | 12 (44.4%) |  |
| No | 20 (54.1%) | 15 (55.6%) |  |
| ACC scores | 7.25 (5.00，8.38) | 6.25 (5.50，8.50) | 0.668 |
| CPB, n (%) |  |  | 0.684 |
| Yes | 4 (10.5%) | 3 (11.1%) |  |
| No | 34 (89.5%) | 24 (88.9%) |  |
| Anesthetic Technique |  |  | 0.317 |
| TIVA | 2 (5.4%) | 0 (0.0%) |  |
| Inhalational anesthesia | 1 (2.7%) | 0 (0.0%) |  |
| Combined Anesthesia | 34 (91.9%) | 27 (100%) |  |
| Surgical duration, h | 2.67 (1.96, 3.83) | 3.00 (1.67，3.92) | 0.591 |
| Anesthesia duration, h | 3.50 (2.92，4.38) | 4.00 (3.25，5.16) | 0.185 |

Supplementary Table1. Baseline and intraoperative characteristics of patients undergoing cardiac surgery. Data are presented as n (%) for categorical variables and median (interquartile range) for continuous variables. Complex cardiac surgery was defined as a RACHS-1 (Risk Adjustment for Congenital Heart Surgery) score >3. ACC: American College of Cardiology; TH, thyroid hormone; ASA, American Society of Anesthesiologists.
